# Current topics in stem cell biology and regenerative medicine: a regional perspective from the United Kingdom

**DOI:** 10.1042/ETLS20210264

**Published:** 2021-10-12

**Authors:** William Eustace Johnson

**Affiliations:** Chester Medical School, University of Chester, Chester, U.K.

**Keywords:** editorial, regenerative medicine, stem cells

## Abstract

This special issue of *Emerging Topics in Life Sciences* entitled ‘Current Topics in Stem Cells and Regenerative Medicine’ brings together expertise from a collaborative organisation known as the Mercia Stem Cell Alliance (MSCA). The alliance was established initially by Professors Sue Kimber (University of Manchester) and Jon Frampton (University of Birmingham) just over 10 years ago and now has multiple regional centres of excellence across the Midlands and North West of the UK, including Aston University, University of Chester, Keele University, Manchester Metropolitan University, Lancaster University, University of Leicester, University of Liverpool, Liverpool John Moore's University, Loughborough University, University of Nottingham, University of Oxford, University of Sheffield, University of York. Many of these centres have contributed reviews to this issue. The MSCA also partners with industrial and clinical organisations, including the NHS, and is active in bringing stem cells and regenerative medicines to a meaningful translational endpoint (see: http://www.msca.manchester.ac.uk/).

The reviews published in the special issue cover key areas in stem cell biology and regenerative medicine, from aspects of basic stem cell research through to the use of stem cells and tissue engineering to model human pathophysiology and for drug development through to clinical applications of adult stem cells and other regenerative medicines. Dogan and Forsyth (Keele University) have reported how epigenetic mechanisms influence telomerase activity, which plays an essential, although not fully understood role in regulating pluripotent stem cell proliferation, differentiation, tissue repair and cancer [1]. Walczak et al. [2] (Aston University) have examined the use of induced pluripotent stem cells and advanced tissue engineering to enable *in vitro* generation of 3D tissue culture models that match the complexity of the human CNS. The concept of using cell spray technologies to deliver stem cells to the CNS in the treatment of neurological trauma and pathology, e.g. following spinal cord injury, has been described and discussed by Chari and co-workers (Keele University) [3]. The use of bio-derived materials as wound dressings to promote skin wound repair has been reviewed by Verdolino et al. [4] (University of Manchester), who also consider how such products have been developed to help overcome inflammatory issues that lead to chronic wounds. There are two complimentary reviews on mesenchymal stem/stromal cells (MSCs). Kuntin and Genever (University of York) have provided an informative historical overview of MSC biology, including MSC differentiation potential and secretome activity, detailing issues of MSC heterogeneity, and calling for further basic research to better understand their mechanisms of action [5]. The need to better understand how MSCs may work as regenerative medicines has been revisited by Amadeo et al. [6] (University of Liverpool), who further critique the application of MSCs in animal disease models, which has contributed to what seems like a confusing picture in terms of determining predictive outcomes for clinical trials in humans. Stewart (Liverpool John Moore's University) has considered the use of adult stem cells and other regenerative medicines, including platelet rich plasma, specifically in the sporting arena. This review demonstrates the potential of stem cells and regenerative medicines to help alleviate human suffering as well as offset financial burdens associated with the treatment costs for traumatic injuries, but also discusses the use of stem cells to enhance sporting performance [7]. Finally, Hulme et al. [8] (RJAH Orthopaedic Hospital/Keele University) review the current state of the art for the use of cell transplants for cartilage repair, which has been a pioneering exemplar in the field of regenerative medicine for musculoskeletal pathology.

We are grateful to *Emerging Topics in Life Sciences* and Portland Press for inviting the reviews, which have been written in such a manner that they are broad in outlook, whilst deep in content. One of the main objectives of the MSCA is to help basic researchers and translational scientists learn and work together, both to increase our fundamental understanding of the molecular and cell biology that underpins this exciting field and also to successfully develop new stem cell therapies. Hence, it is hoped that the special issue will provide a useful resource to many current and next generation investigators as they progress in their careers in stem cell research.

## Abbreviations

MSCA: Mercia Stem Cell Alliance
MSCs: mesenchymal stem/stromal cells
